# Primary small cell neuroendocrine carcinoma of the urinary bladder with coexisting high-grade urothelial carcinoma: a case report and a review of the literature

**DOI:** 10.1186/1742-6413-2-18

**Published:** 2005-11-04

**Authors:** Marilyn Bui, Walid E Khalbuss

**Affiliations:** 1 Department of Interdisciplinary Oncology, Division of Anatomical Pathology, H. Lee Moffitt Cancer Center and Research Institute, Tampa, Florida, USA; 2 Department of Pathology, University of Florida Health Science Center (UFHSC), Jacksonville, Florida, USA

**Keywords:** urinary cytology, urinary bladder, small cell carcinoma, urothelial carcinoma

## Abstract

Primary neuroendocrine carcinomas of the urinary bladder are rare. Here, we report a case of an 82-year-old man who presented with hematuria and was found to have an ulcerated lesion in the bladder. A diagnosis of small neuroendocrine cell carcinoma with coexisting minor high-grade urothelial components was rendered. In this report, the clinical, cytological, histological, and immunohistochemical features of this case are described, and a review of the literature about this neoplasm is presented. The differential diagnoses of small cell tumor in urinary bladder washing specimens are discussed.

## 

Neuroendocrine carcinoma comprises carcinoid tumors, large cell neuroendocrine carcinomas, and small cell carcinomas. Primary neuroendocrine carcinomas of the urinary bladder are rare. They usually involve male patients and coexist with urothelial carcinoma [1]. Among neuroendocrine tumors of the urinary bladder, small cell carcinomas are most common with more than 100 cases having been described [2-4]; carcinoid tumors are much less common; and large cell neuroendocrine carcinomas are very rare with only 3 cases reported [5]. The rare nature of a primary neuroendocrine carcinoma, especially in a cytology specimen, should be included in consideration as part of the differential diagnosis of the far more common urothelial carcinoma.

## Case Reports

The patient was an 82-year-old male who presented with hematuria for 3 months. Cystoscopy revealed an ulcerated lesion in the bladder trigone. A bladder washing and a bladder biopsy were performed. The patient underwent bladder tumor resection and subsequent cystoprostatectomy. The patient's recovery was complicated by gastrointestinal bleeding, and he eventually expired within 3 months of his initial diagnosis. Autopsy concluded that the patient died from extensive upper gastrointestinal bleeding secondary to small bowel involvement with metastatic small cell carcinoma superimposed by DIC.

**Figure 1:**
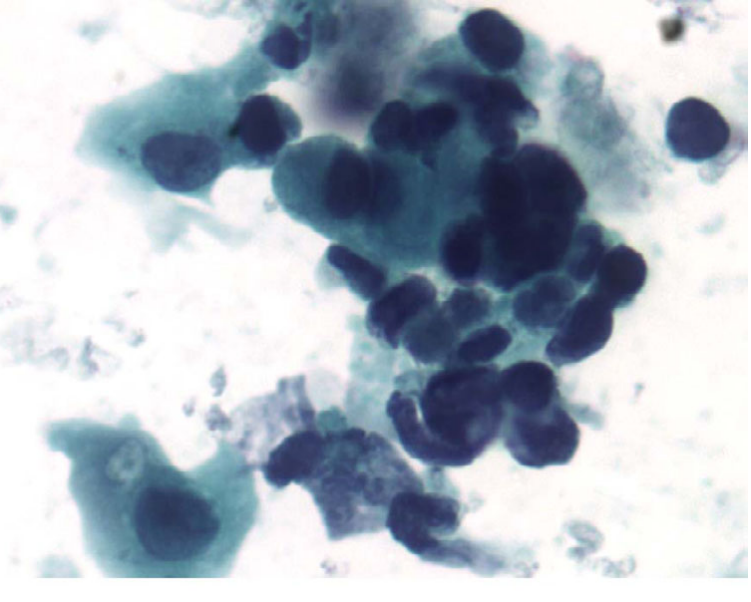
Bladder washing, cytospin preparation, Papanicolaou stain, ×400. Mixed small undifferentiated carcinoma and large urothelial carcinoma cells. The small cells are seen singly and in clusters with scanty cytoplasm, moderate cellular pleomorphism, nuclear molding, dark chromatin, irregular nuclear contour, and background of blood and necrosis consistent with a small cell carcinoma. In addition, there are a few atypical cells with a moderate amount of cytoplasm, small nucleoli, and irregular nuclear contours consistent with high-grade urothelial carcinoma. Figure 2 inset: comparison of two small cell carcinoma cells and one large urothelial carcinoma cell.

**Figure 2:**
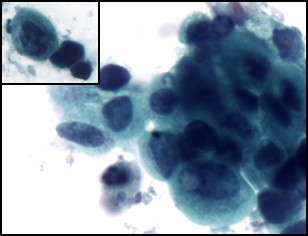
Bladder washing, cytospin preparation, Papanicolaou stain, ×400. Mixed small undifferentiated carcinoma and large urothelial carcinoma cells. The small cells are seen singly and in clusters with scanty cytoplasm, moderate cellular pleomorphism, nuclear molding, dark chromatin, irregular nuclear contour, and background of blood and necrosis consistent with a small cell carcinoma. In addition, there are a few atypical cells with a moderate amount of cytoplasm, small nucleoli, and irregular nuclear contours consistent with high-grade urothelial carcinoma. Figure 2 inset: comparison of two small cell carcinoma cells and one large urothelial carcinoma cell.

**Figure 3:**
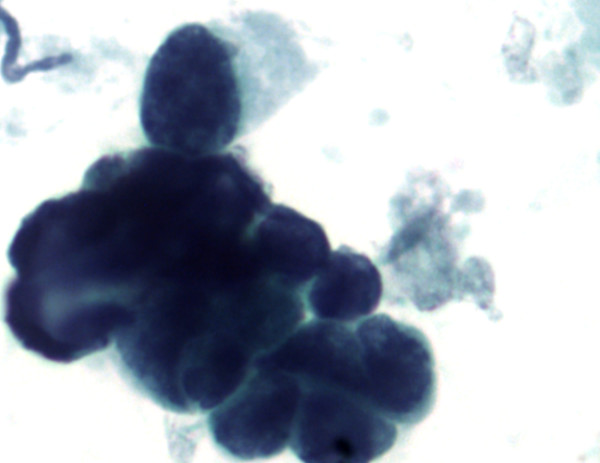
Bladder washing, cell block, H&E, ×400: clusters of pure, small cell undifferentiated carcinoma of the bladder. The small cell carcinoma cells are approximately twice the size of the RBCs with finely granular chromatin and nuclear molding.

**Figure 4:**
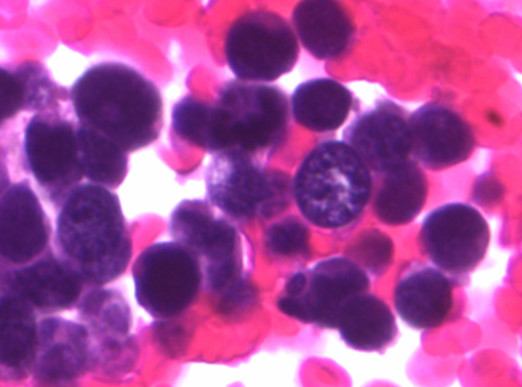
Bladder washing, cell block, H&E, ×400: clusters of pure, small cell undifferentiated carcinoma of the bladder. The small cell carcinoma cells are approximately twice the size of the RBCs with finely granular chromatin and nuclear molding.

The cytological features of cytospin smears and cellblock of the bladder washing are presented in Figures 1, 2, 3, 4, &6. The specimen was hypercellular and consisted of highly atypical cells with two distinct populations: small cells and large cells.

**Figure 6:**
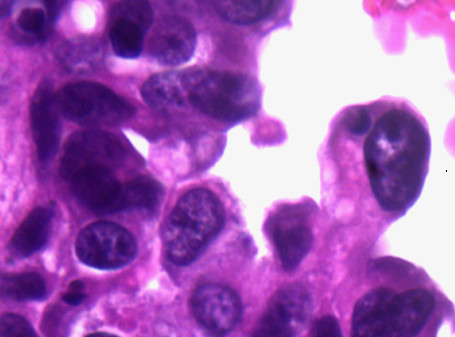
Cell block, H&E, ×400: Urothelial carcinoma cells with a moderate amount of cytoplasm and small nucleoli.

The small cell population was the predominant one. It showed undifferentiated malignant small cells ranging in size and shape from lymphocyte-like to intermediate type (see Figure 4 for comparison with RBC size). The cells demonstrated a moderate degree of cellular pleomorphism, nuclear molding, finely granular chromatin, hyperchromatic nuclei, and inconspicuous nucleoli in a bloody and necrotic background (Figures 1, 2, 3). Occasional elongated cells and occasional rosette formation were also noted. These cytomorphologic features were indicative of small undifferentiated cell carcinoma. The cellblock (Figure 4) findings recapitulated the findings in the smears and were useful in subsequent immunohistochemical evaluations.

The large cell population was a minor component and showed highly atypical cells with central nuclei, small nucleoli, irregular nuclear contour, and dense cytoplasm in a bloody and necrotic background (Figures 1 &2). These cytomorphologic features were indicative of a high-grade urothelial carcinoma component. The cell block (Figure 6) findings recapitulated the findings in the smears and were useful in subsequent immunohistochemical evaluations.

**Figure 5:**
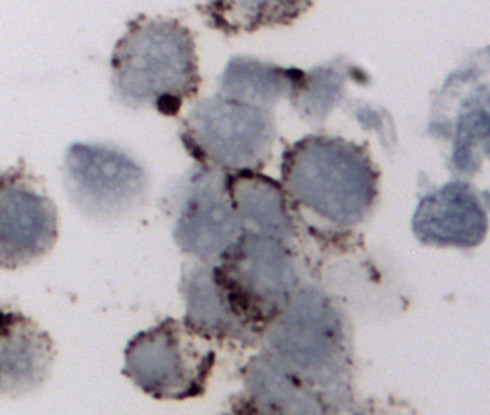
Bladder washing, cell block, synaptophysin immunostain of small cell carcinoma, ×400. The tumor cells demonstrated cytoplasmic positivity.

Immunohistochemical stains were performed on cell blocks and showed positive staining of the small cell carcinoma component only for synaptophysin (Figure 5). The diagnosis was rendered as small cell neuroendocrine carcinoma with a high-grade urothelial carcinoma component.

**Figure 7:**
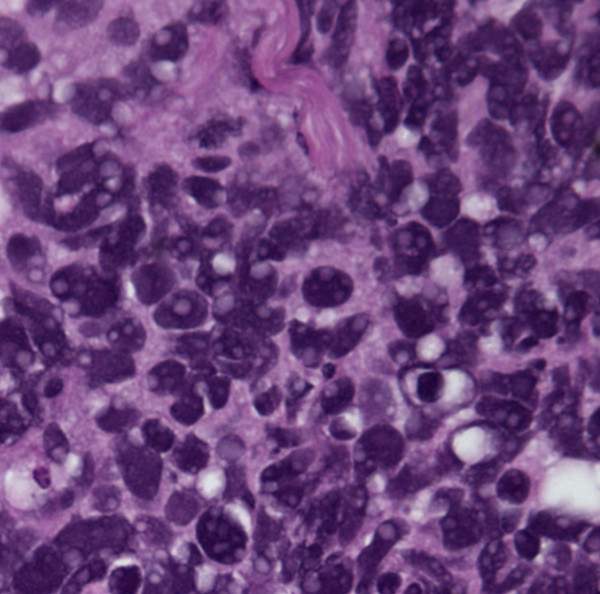
Tissue biopsy of the bladder tumor, H&E, ×200. Figure 7; Small cell carcinoma. Figure 8; Urothelial carcinoma with active mitosis evident.

**Figure 8:**
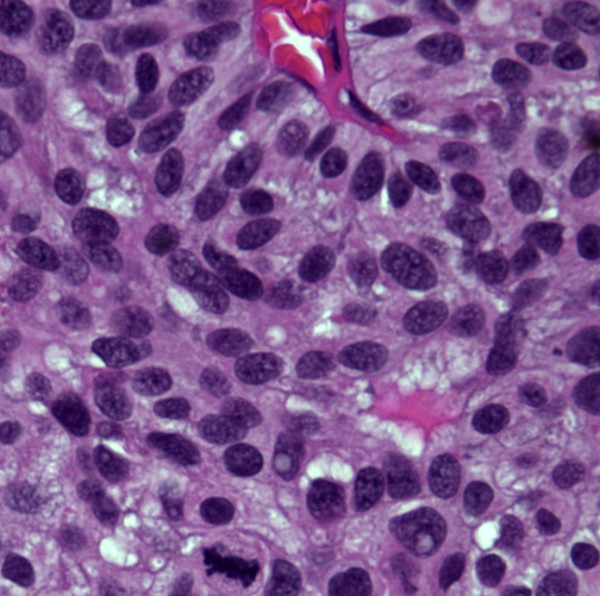
Tissue biopsy of the bladder tumor, H&E, ×200. Figure 7; Small cell carcinoma. Figure 8; Urothelial carcinoma with active mitosis evident.

**Figure 9:**
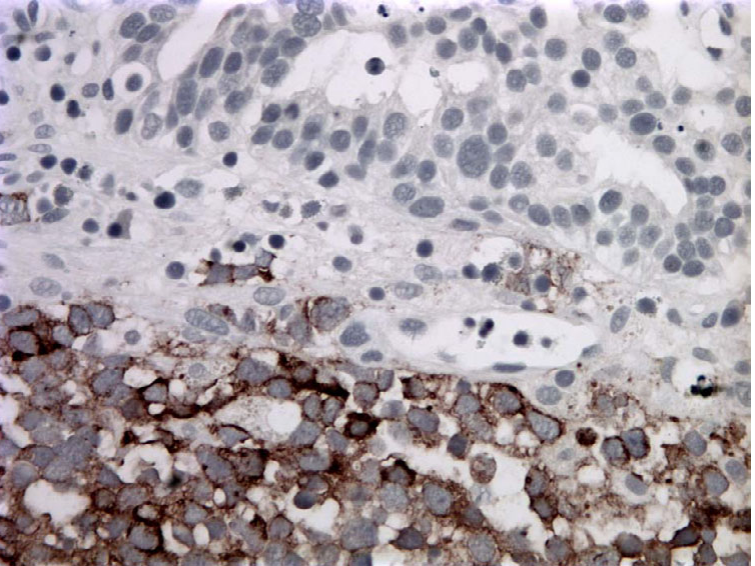
Tissue biopsy of the bladder tumor, synaptophysin immunostain. The bottom half of the section is the small cell carcinoma component of the bladder tumor which demonstrated synaptophysin positivity. The top half of the section is urothelial carcinoma that is negative for synaptophysin.

Subsequently, the patient underwent bladder tumor resection and a 4 cm tumor was resected. The histology sections (Figures 7 and 8) demonstrated a small cell carcinoma in about 80% of the tumor (Figure 7) and a high-grade urothelial carcinoma in about 20% of the tumor (Figure 8). Immunohistochemical stains were performed and the tumor cells of the small cell carcinoma component were positive selectively for synaptophysin (Figure 9) and chromogranin, whereas, the high-grade urothelial carcinoma stained selectively for CK7, and CK20.

A subsequent radical cystectomy revealed a residual tumor of 4 cm in the greatest dimension. The tumor also appeared to have invaded through the muscularis propria into the adjacent fat. There were fifteen out of twenty-one bilateral lymph nodes involved by the small cell undifferentiated carcinoma. The tumor stage was yT_3_N_2 _Mx.

The autopsy revealed an intra-abdominal mass showing extensive metastatic small cell carcinoma (18 cm) with involvement of the duodenum (the entire duodenal wall including the mucosa) with extensive adhesions to the retroperitoneum, descending aorta, periaortic lymph nodes, and mesenteric fat. Tumor also involved peripancreatic and peri adrenal adipose tissue. Autopsy concluded that the patient died from extensive upper gastrointestinal bleeding secondary to small bowel involvement with metastatic small cell carcinoma superimposed by DIC.

## Discussion

Here, we present a case of primary small cell carcinoma of the urinary bladder coexisting with a high-grade urothelial carcinoma component. The cytomorphological pattern of the bladder washing was that of a small, blue cell neoplasm. The differential diagnoses included a high-grade urothelial carcinoma, a small cell carcinoma of the urinary bladder, and a lymphoma, as well as metastatic lesions, especially from the lung. Lymphomas of the bladder are rare, and have a good prognosis with a good response to chemotherapy [6]. The cytomorphological features of bladder lymphoma include single pleomorphic cells with round to oval nuclei, increased nuclear/cytoplasmic ratio, and centrally located nuclei often with prominent nucleoli [7]. The cytomorphological features of metastatic small cell carcinoma of lung origin are indistinguishable from primary small cell carcinoma of the bladder. However, the presence of a high-grade urothelial carcinoma component in our case was suggestive evidence of a primary bladder lesion. In conjunction with the patient's clinical presentation and clinical work-up, the cytomorphology and immunocytochemical features supported the diagnosis of a primary small cell carcinoma of the urinary bladder. Subsequent surgical excision revealed a high-grade urothelial carcinoma and small cell carcinoma.

Making the diagnosis of a small cell carcinoma of the urinary bladder on a cytology specimen is important. Primary neuroendocrine carcinomas of the bladder usually are at an advanced stage at presentation. As in this case, the patient already had local invasion and lymph node metastases at the time of diagnosis. Although bladder neuroendocrine carcinoma is an aggressive tumor, the prognosis is better than those patients with neuroendocrine carcinoma of other sites [8].

## References

[B1] Abenoza P, Manivel C, Siblry R (1986). Adenocarcinoma with neuroendocrine differentiation of the urinaryl bladder. Arch Pathol Lab Me.

[B2] Mills S, Wolfe J, Weiss M (1989). Small cell undifferentialted carcinoma of the urinary bladder: a light-microscopic, immunochemical, and ultrastructureal study of 12 cases. Am J Surg Pathol.

[B3] Grignon D, Ro J, Ayala A (1992). Small cell carcinoma of the urinary bladder: a clinicopathologic analysis of 22 cases. Cancer.

[B4] Ali S, Reuter V, Zakowski M (1997). Small cell neuroendocrine carcinoma of the urinary bladder: a clinicopathologic study with emphasis on cytologic features. Cancer.

[B5] Evans AJ, Al-Maghrabi J, Tsihlias J, Lajoie G, Sweet JM, Chapman WI (2002). Primary large cell neuroendocrine carcinoma of the urinary bladder. Arch J Pathol Lab Med.

[B6] Leite KR, Bruschini H, Camara-Lopes LH (2004). Primary lymphoma of the bladder. Int Braz J Urol.

[B7] Quinn AM, Flanigan R, Sienko A, Wojcik EM (2004). Cytologic features of recurrent lymphoma involving the urinary bladder. Diagn Cytopathol.

[B8] Holmang S, Borghede G, Johansson SL (1995). Primary small cell carcinoma of the bladder: a report of 25 cases. J Urol.

